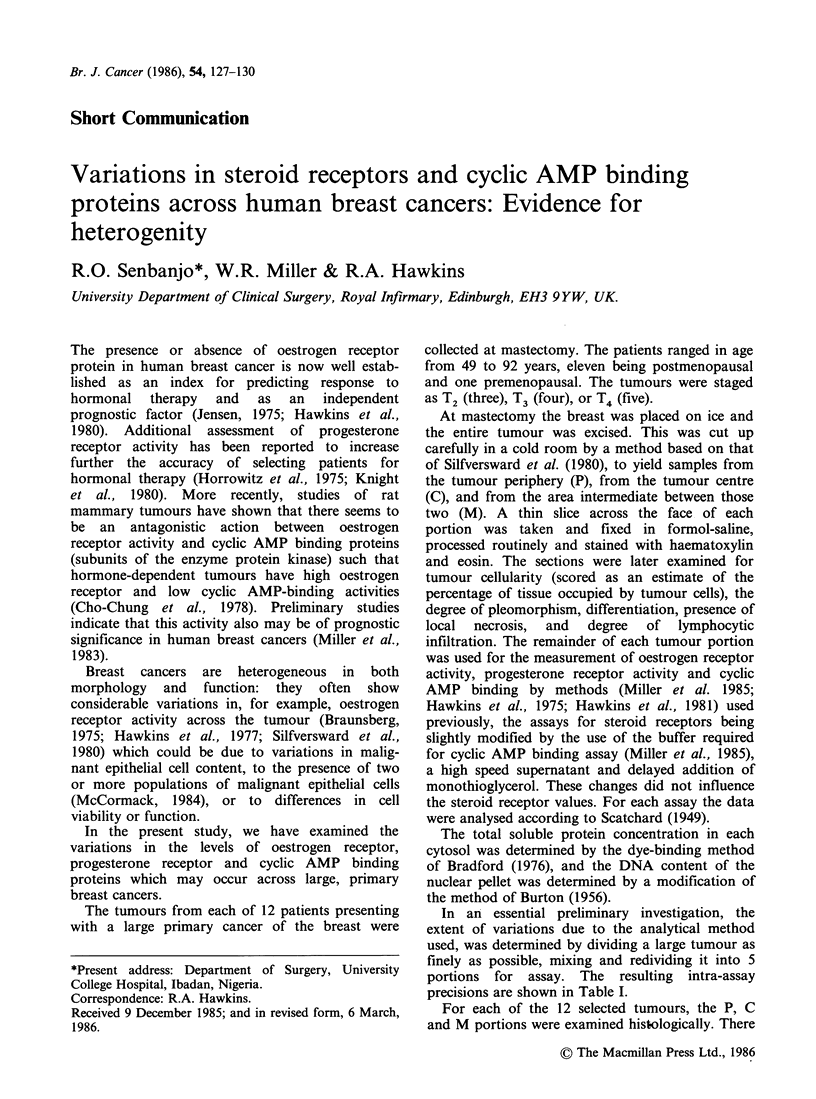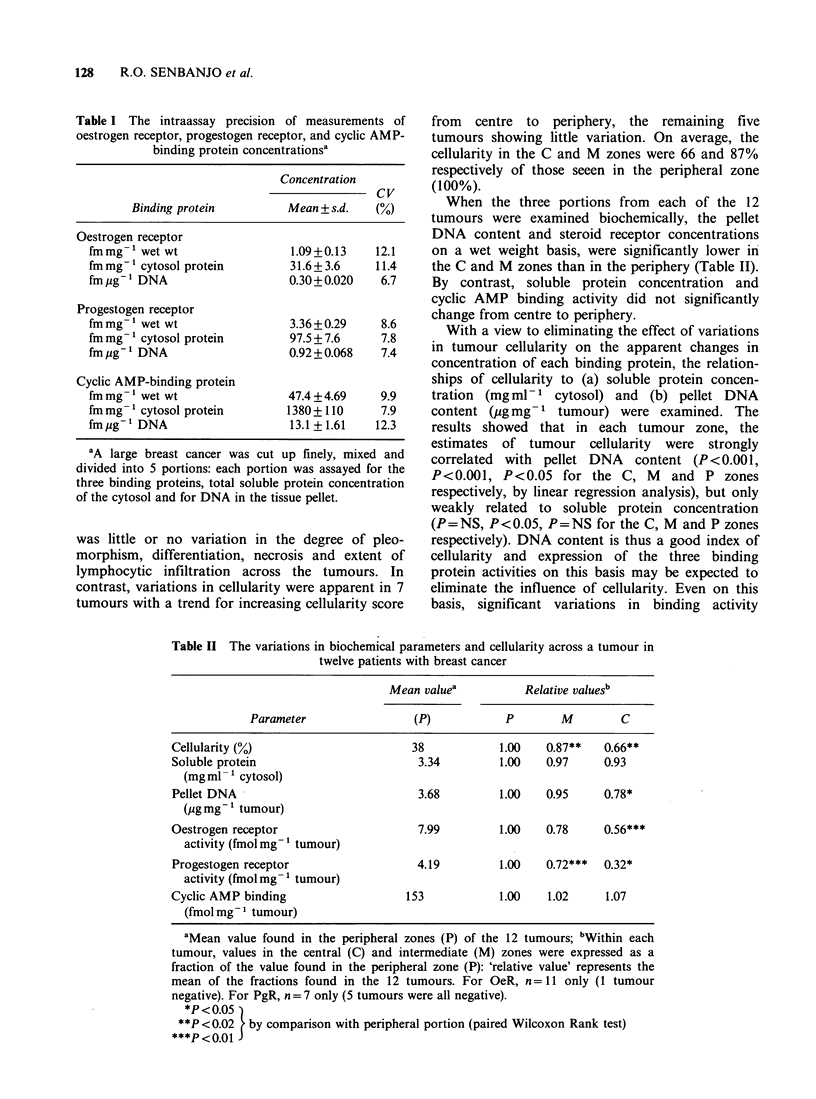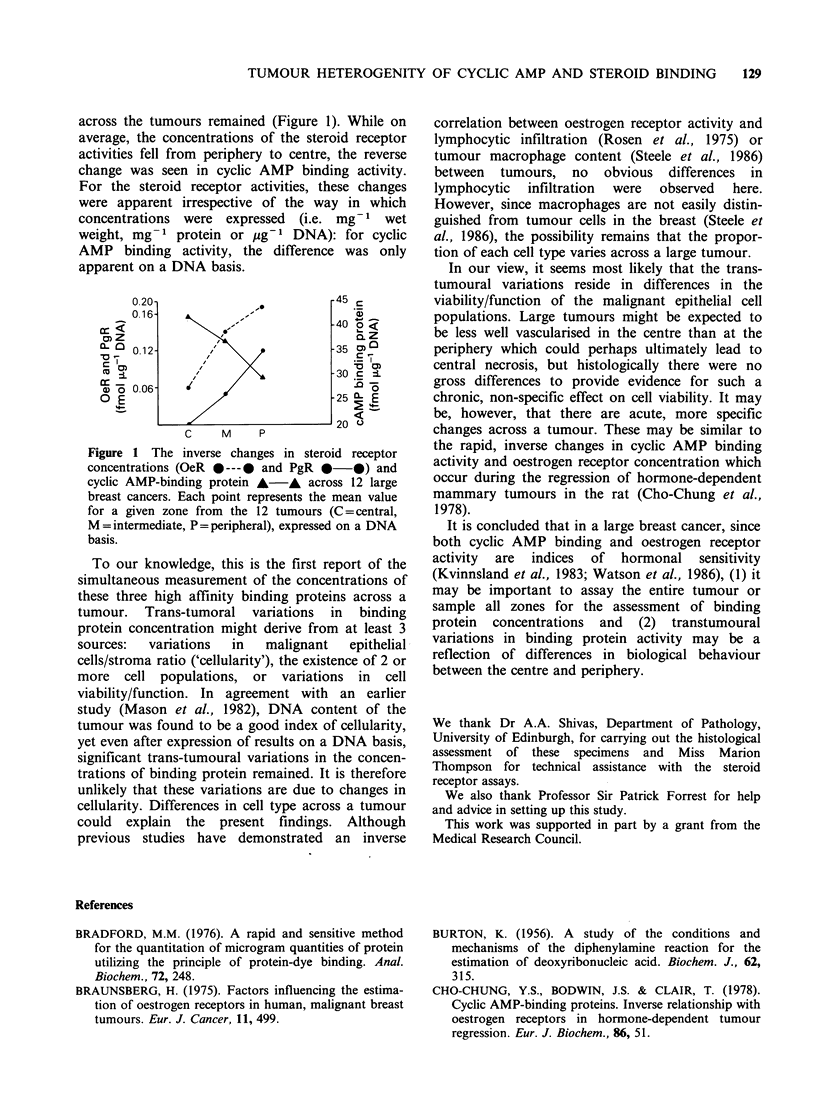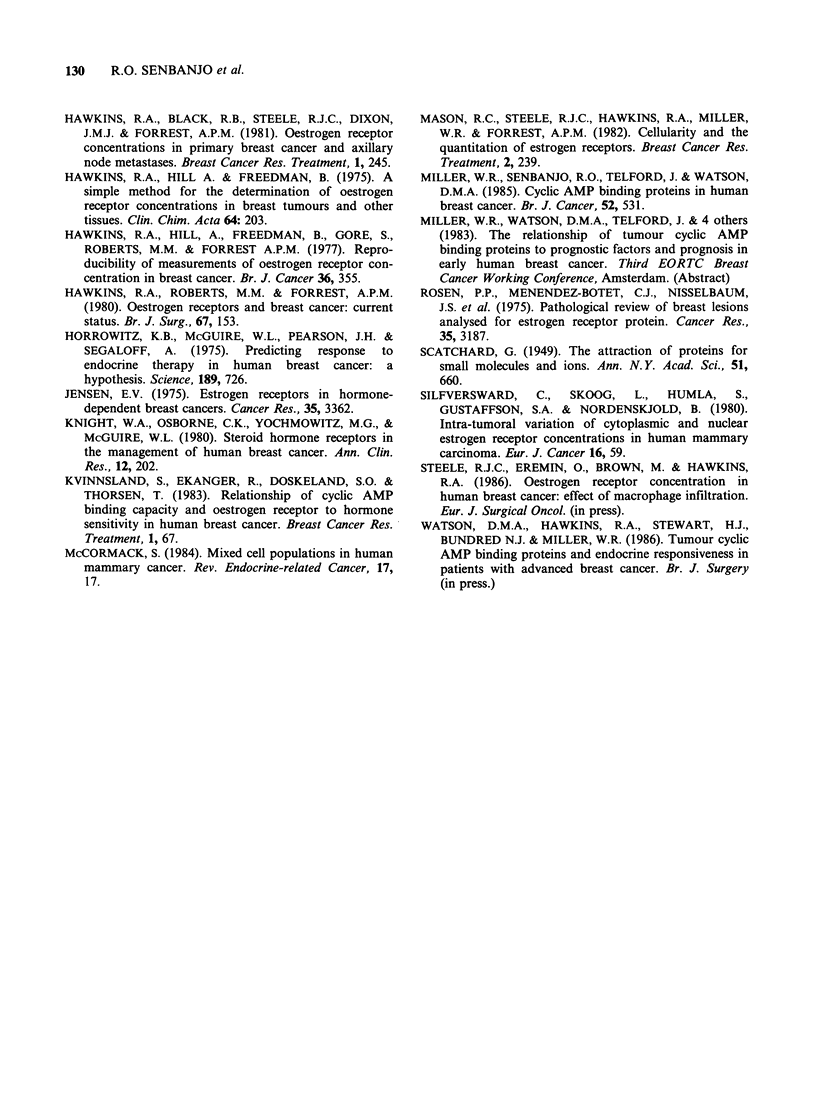# Variations in steroid receptors and cyclic AMP binding proteins across human breast cancers: evidence for heterogeneity.

**DOI:** 10.1038/bjc.1986.162

**Published:** 1986-07

**Authors:** R. O. Senbanjo, W. R. Miller, R. A. Hawkins


*Br. J. Cancer* (1986), **54**, 127–130

### Short Communication

### Variations in steroid receptors and cyclic AMP binding proteins across human breast cancers: Evidence for heterogenity

R.O. Senbanjo*, W.R. Miller & R.A. Hawkins

*University Department of Clinical Surgery, Royal Infirmary, Edinburgh, EH3 9YW, UK.*

The presence or absence of oestrogen receptor protein in human breast cancer is now well established as an index for predicting response to hormonal therapy and as an independent prognostic factor (Jensen, 1975; Hawkins *et al.*, 1980). Additional assessment of progesterone receptor activity has been reported to increase further the accuracy of selecting patients for hormonal therapy (Horrowitz *et al.*, 1975; Knight *et al.*, 1980). More recently, studies of rat mammary tumours have shown that there seems to be an antagonistic action between oestrogen receptor activity and cyclic AMP binding proteins (subunits of the enzyme protein kinase) such that hormone-dependent tumours have high oestrogen receptor and low cyclic AMP-binding activities (Cho-Chung *et al.*, 1978). Preliminary studies indicate that this activity also may be of prognostic significance in human breast cancers (Miller *et al.*, 1983).

Breast cancers are heterogeneous in both morphology and function: they often show considerable variations in, for example, oestrogen receptor activity across the tumour (Braunsberg, 1975; Hawkins *et al.*, 1977; Silfversward *et al.*, 1980) which could be due to variations in malignant epithelial cell content, to the presence of two or more populations of malignant epithelial cells (McCormack, 1984), or to differences in cell viability or function.

In the present study, we have examined the variations in the levels of oestrogen receptor, progesterone receptor and cyclic AMP binding proteins which may occur across large, primary breast cancers.

The tumours from each of 12 patients presenting with a large primary cancer of the breast were collected at mastectomy. The patients ranged in age from 49 to 92 years, eleven being postmenopausal and one premenopausal. The tumours were staged as $T_2$ (three), $T_3$ (four), or $T_4$ (five).

At mastectomy the breast was placed on ice and the entire tumour was excised. This was cut up carefully in a cold room by a method based on that of Silfversward *et al.* (1980), to yield samples from the tumour periphery (P), from the tumour centre (C), and from the area intermediate between those two (M). A thin slice across the face of each portion was taken and fixed in formol-saline, processed routinely and stained with haematoxylin and eosin. The sections were later examined for tumour cellularity (scored as an estimate of the percentage of tissue occupied by tumour cells), the degree of pleomorphism, differentiation, presence of local necrosis, and degree of lymphocytic infiltration. The remainder of each tumour portion was used for the measurement of oestrogen receptor activity, progesterone receptor activity and cyclic AMP binding by methods (Miller *et al.* 1985; Hawkins *et al.*, 1975; Hawkins *et al.*, 1981) used previously, the assays for steroid receptors being slightly modified by the use of the buffer required for cyclic AMP binding assay (Miller *et al.*, 1985), a high speed supernatant and delayed addition of monothioglycerol. These changes did not influence the steroid receptor values. For each assay the data were analysed according to Scatchard (1949).

The total soluble protein concentration in each cytosol was determined by the dye-binding method of Bradford (1976), and the DNA content of the nuclear pellet was determined by a modification of the method of Burton (1956).

In an essential preliminary investigation, the extent of variations due to the analytical method used, was determined by dividing a large tumour as finely as possible, mixing and redividing it into 5 portions for assay. The resulting intra-assay precisions are shown in Table I.

For each of the 12 selected tumours, the P, C and M portions were examined histologically. There

*Present address: Department of Surgery, University College Hospital, Ibadan, Nigeria.
Correspondence: R.A. Hawkins.
Received 9 December 1985; and in revised form, 6 March, 1986.

© The Macmillan Press Ltd., 1986

**Table I** The intraassay precision of measurements of oestrogen receptor, progestogen receptor, and cyclic AMP-binding protein concentrations[a]

| Binding protein | Concentration Mean ± s.d. | CV (%) |
|---|---|---|
| Oestrogen receptor | | |
| fm mg$^{-1}$ wet wt | 1.09 ± 0.13 | 12.1 |
| fm mg$^{-1}$ cytosol protein | 31.6 ± 3.6 | 11.4 |
| fm $\mu$g$^{-1}$ DNA | 0.30 ± 0.020 | 6.7 |
| Progestogen receptor | | |
| fm mg$^{-1}$ wet wt | 3.36 ± 0.29 | 8.6 |
| fm mg$^{-1}$ cytosol protein | 97.5 ± 7.6 | 7.8 |
| fm $\mu$g$^{-1}$ DNA | 0.92 ± 0.068 | 7.4 |
| Cyclic AMP-binding protein | | |
| fm mg$^{-1}$ wet wt | 47.4 ± 4.69 | 9.9 |
| fm mg$^{-1}$ cytosol protein | 1380 ± 110 | 7.9 |
| fm $\mu$g$^{-1}$ DNA | 13.1 ± 1.61 | 12.3 |

[a]A large breast cancer was cut up finely, mixed and divided into 5 portions: each portion was assayed for the three binding proteins, total soluble protein concentration of the cytosol and for DNA in the tissue pellet.

was little or no variation in the degree of pleomorphism, differentiation, necrosis and extent of lymphocytic infiltration across the tumours. In contrast, variations in cellularity were apparent in 7 tumours with a trend for increasing cellularity score from centre to periphery, the remaining five tumours showing little variation. On average, the cellularity in the C and M zones were 66 and 87% respectively of those seeen in the peripheral zone (100%).

When the three portions from each of the 12 tumours were examined biochemically, the pellet DNA content and steroid receptor concentrations on a wet weight basis, were significantly lower in the C and M zones than in the periphery (Table II). By contrast, soluble protein concentration and cyclic AMP binding activity did not significantly change from centre to periphery.

With a view to eliminating the effect of variations in tumour cellularity on the apparent changes in concentration of each binding protein, the relationships of cellularity to (a) soluble protein concentration (mg ml$^{-1}$ cytosol) and (b) pellet DNA content ($\mu$g mg$^{-1}$ tumour) were examined. The results showed that in each tumour zone, the estimates of tumour cellularity were strongly correlated with pellet DNA content ($P<0.001$, $P<0.001$, $P<0.05$ for the C, M and P zones respectively, by linear regression analysis), but only weakly related to soluble protein concentration ($P=$NS, $P<0.05$, $P=$NS for the C, M and P zones respectively). DNA content is thus a good index of cellularity and expression of the three binding protein activities on this basis may be expected to eliminate the influence of cellularity. Even on this basis, significant variations in binding activity

**Table II** The variations in biochemical parameters and cellularity across a tumour in twelve patients with breast cancer

| Parameter | Mean value[a] (P) | Relative values[b] P | M | C |
|---|---|---|---|---|
| Cellularity (%) | 38 | 1.00 | 0.87** | 0.66** |
| Soluble protein (mg ml$^{-1}$ cytosol) | 3.34 | 1.00 | 0.97 | 0.93 |
| Pellet DNA ($\mu$g mg$^{-1}$ tumour) | 3.68 | 1.00 | 0.95 | 0.78* |
| Oestrogen receptor activity (fmol mg$^{-1}$ tumour) | 7.99 | 1.00 | 0.78 | 0.56*** |
| Progestogen receptor activity (fmol mg$^{-1}$ tumour) | 4.19 | 1.00 | 0.72*** | 0.32* |
| Cyclic AMP binding (fmol mg$^{-1}$ tumour) | 153 | 1.00 | 1.02 | 1.07 |

[a]Mean value found in the peripheral zones (P) of the 12 tumours; [b]Within each tumour, values in the central (C) and intermediate (M) zones were expressed as a fraction of the value found in the peripheral zone (P): 'relative value' represents the mean of the fractions found in the 12 tumours. For OeR, $n=11$ only (1 tumour negative). For PgR, $n=7$ only (5 tumours were all negative).

*$P<0.05$
**$P<0.02$ } by comparison with peripheral portion (paired Wilcoxon Rank test)
***$P<0.01$

across the tumours remained (Figure 1). While on average, the concentrations of the steroid receptor activities fell from periphery to centre, the reverse change was seen in cyclic AMP binding activity. For the steroid receptor activities, these changes were apparent irrespective of the way in which concentrations were expressed (i.e. $mg^{-1}$ wet weight, $mg^{-1}$ protein or $\mu g^{-1}$ DNA): for cyclic AMP binding activity, the difference was only apparent on a DNA basis.

**Figure 1** The inverse changes in steroid receptor concentrations (OeR ●---● and PgR ●——●) and cyclic AMP-binding protein ▲——▲ across 12 large breast cancers. Each point represents the mean value for a given zone from the 12 tumours (C = central, M = intermediate, P = peripheral), expressed on a DNA basis.

To our knowledge, this is the first report of the simultaneous measurement of the concentrations of these three high affinity binding proteins across a tumour. Trans-tumoral variations in binding protein concentration might derive from at least 3 sources: variations in malignant epithelial cells/stroma ratio ('cellularity'), the existence of 2 or more cell populations, or variations in cell viability/function. In agreement with an earlier study (Mason *et al.*, 1982), DNA content of the tumour was found to be a good index of cellularity, yet even after expression of results on a DNA basis, significant trans-tumoural variations in the concentrations of binding protein remained. It is therefore unlikely that these variations are due to changes in cellularity. Differences in cell type across a tumour could explain the present findings. Although previous studies have demonstrated an inverse correlation between oestrogen receptor activity and lymphocytic infiltration (Rosen *et al.*, 1975) or tumour macrophage content (Steele *et al.*, 1986) between tumours, no obvious differences in lymphocytic infiltration were observed here. However, since macrophages are not easily distinguished from tumour cells in the breast (Steele *et al.*, 1986), the possibility remains that the proportion of each cell type varies across a large tumour.

In our view, it seems most likely that the trans-tumoural variations reside in differences in the viability/function of the malignant epithelial cell populations. Large tumours might be expected to be less well vascularised in the centre than at the periphery which could perhaps ultimately lead to central necrosis, but histologically there were no gross differences to provide evidence for such a chronic, non-specific effect on cell viability. It may be, however, that there are acute, more specific changes across a tumour. These may be similar to the rapid, inverse changes in cyclic AMP binding activity and oestrogen receptor concentration which occur during the regression of hormone-dependent mammary tumours in the rat (Cho-Chung *et al.*, 1978).

It is concluded that in a large breast cancer, since both cyclic AMP binding and oestrogen receptor activity are indices of hormonal sensitivity (Kvinnsland *et al.*, 1983; Watson *et al.*, 1986), (1) it may be important to assay the entire tumour or sample all zones for the assessment of binding protein concentrations and (2) transtumoural variations in binding protein activity may be a reflection of differences in biological behaviour between the centre and periphery.

We thank Dr A.A. Shivas, Department of Pathology, University of Edinburgh, for carrying out the histological assessment of these specimens and Miss Marion Thompson for technical assistance with the steroid receptor assays.

We also thank Professor Sir Patrick Forrest for help and advice in setting up this study.

This work was supported in part by a grant from the Medical Research Council.

### References

BRADFORD, M.M. (1976). A rapid and sensitive method for the quantitation of microgram quantities of protein utilizing the principle of protein-dye binding. *Anal. Biochem.*, **72**, 248.

BRAUNSBERG, H. (1975). Factors influencing the estimation of oestrogen receptors in human, malignant breast tumours. *Eur. J. Cancer*, **11**, 499.

BURTON, K. (1956). A study of the conditions and mechanisms of the diphenylamine reaction for the estimation of deoxyribonucleic acid. *Biochem. J.*, **62**, 315.

CHO-CHUNG, Y.S., BODWIN, J.S. & CLAIR, T. (1978). Cyclic AMP-binding proteins. Inverse relationship with oestrogen receptors in hormone-dependent tumour regression. *Eur. J. Biochem.*, **86**, 51.

HAWKINS, R.A., BLACK, R.B., STEELE, R.J.C., DIXON, J.M.J. & FORREST, A.P.M. (1981). Oestrogen receptor concentrations in primary breast cancer and axillary node metastases. *Breast Cancer Res. Treatment,* **1,** 245.

HAWKINS, R.A., HILL A. & FREEDMAN, B. (1975). A simple method for the determination of oestrogen receptor concentrations in breast tumours and other tissues. *Clin. Chim. Acta* **64:** 203.

HAWKINS, R.A., HILL, A., FREEDMAN, B., GORE, S., ROBERTS, M.M. & FORREST A.P.M. (1977). Reproducibility of measurements of oestrogen receptor concentration in breast cancer. *Br. J. Cancer* **36,** 355.

HAWKINS, R.A., ROBERTS, M.M. & FORREST, A.P.M. (1980). Oestrogen receptors and breast cancer: current status. *Br. J. Surg.,* **67,** 153.

HORROWITZ, K.B., McGUIRE, W.L., PEARSON, J.H. & SEGALOFF, A. (1975). Predicting response to endocrine therapy in human breast cancer: a hypothesis. *Science,* **189,** 726.

JENSEN, E.V. (1975). Estrogen receptors in hormone-dependent breast cancers. *Cancer Res.,* **35,** 3362.

KNIGHT, W.A., OSBORNE, C.K., YOCHMOWITZ, M.G., & McGUIRE, W.L. (1980). Steroid hormone receptors in the management of human breast cancer. *Ann. Clin. Res.,* **12,** 202.

KVINNSLAND, S., EKANGER, R., DOSKELAND, S.O. & THORSEN, T. (1983). Relationship of cyclic AMP binding capacity and oestrogen receptor to hormone sensitivity in human breast cancer. *Breast Cancer Res. Treatment,* **1,** 67.

McCORMACK, S. (1984). Mixed cell populations in human mammary cancer. *Rev. Endocrine-related Cancer,* **17,** 17.

MASON, R.C., STEELE, R.J.C., HAWKINS, R.A., MILLER, W.R. & FORREST, A.P.M. (1982). Cellularity and the quantitation of estrogen receptors. *Breast Cancer Res. Treatment,* **2,** 239.

MILLER, W.R., SENBANJO, R.O., TELFORD, J. & WATSON, D.M.A. (1985). Cyclic AMP binding proteins in human breast cancer. *Br. J. Cancer,* **52,** 531.

MILLER, W.R., WATSON, D.M.A., TELFORD, J. & 4 others (1983). The relationship of tumour cyclic AMP binding proteins to prognostic factors and prognosis in early human breast cancer. *Third EORTC Breast Cancer Working Conference,* Amsterdam. (Abstract)

ROSEN, P.P., MENENDEZ-BOTET, C.J., NISSELBAUM, J.S. *et al.* (1975). Pathological review of breast lesions analysed for estrogen receptor protein. *Cancer Res.,* **35,** 3187.

SCATCHARD, G. (1949). The attraction of proteins for small molecules and ions. *Ann. N.Y. Acad. Sci.,* **51,** 660.

SILFVERSWARD, C., SKOOG, L., HUMLA, S., GUSTAFFSON, S.A. & NORDENSKJOLD, B. (1980). Intra-tumoral variation of cytoplasmic and nuclear estrogen receptor concentrations in human mammary carcinoma. *Eur. J. Cancer* **16,** 59.

STEELE, R.J.C., EREMIN, O., BROWN, M. & HAWKINS, R.A. (1986). Oestrogen receptor concentration in human breast cancer: effect of macrophage infiltration. *Eur. J. Surgical Oncol.* (in press).

WATSON, D.M.A., HAWKINS, R.A., STEWART, H.J., BUNDRED N.J. & MILLER, W.R. (1986). Tumour cyclic AMP binding proteins and endocrine responsiveness in patients with advanced breast cancer. *Br. J. Surgery* (in press.)